# Very low HDL levels: clinical assessment and management

**DOI:** 10.20945/2359-3997000000585

**Published:** 2023-01-01

**Authors:** Isabella Bonilha, Beatriz Luchiari, Wilson Nadruz, Andrei C. Sposito

**Affiliations:** 1 Universidade de Campinas Laboratório de Biologia Vascular e Aterosclerose (AtheroLab) Divisão de Cardiologia Campinas SP Brasil Universidade de Campinas (Unicamp), Laboratório de Biologia Vascular e Aterosclerose (AtheroLab), Divisão de Cardiologia, Campinas, SP, Brasil; 2 Universidade de Campinas Divisão de Cardiologia Campinas SP Brasil Universidade de Campinas (Unicamp), Divisão de Cardiologia, Campinas, SP, Brasil

**Keywords:** High-density lipoprotein, Tangier disease, LCAT deficiency, familial hypoalphalipoproteinemia, polygenic dyslipidemias, atherosclerosis

## Abstract

In individuals with very low high-density lipoprotein (HDL-C) cholesterol, such as Tangier disease, LCAT deficiency, and familial hypoalphalipoproteinemia, there is an increased risk of premature atherosclerosis. However, analyzes based on comparisons of populations with small variations in HDL-C mediated by polygenic alterations do not confirm these findings, suggesting that there is an indirect association or heterogeneity in the pathophysiological mechanisms related to the reduction of HDL-C. Trials that evaluated some of the HDL functions demonstrate a more robust degree of association between the HDL system and atherosclerotic risk, but as they were not designed to modify lipoprotein functionality, there is insufficient data to establish a causal relationship. We currently have randomized clinical trials of therapies that increase HDL-C concentration by various mechanisms, and this HDL-C elevation has not independently demonstrated a reduction in the risk of cardiovascular events. Therefore, this evidence shows that (a) measuring HDL-C as a way of estimating HDL-related atheroprotective system function is insufficient and (b) we still do not know how to increase cardiovascular protection with therapies aimed at modifying HDL metabolism. This leads us to a greater effort to understand the mechanisms of molecular action and cellular interaction of HDL, completely abandoning the traditional view focused on the plasma concentration of HDL-C. In this review, we will detail this new understanding and the new horizon for using the HDL system to mitigate residual atherosclerotic risk.

## INTRODUCTION

Important findings from the Framingham (1), Tromsø Heart (2) and Prospective Cardiovascular Munster (PROCAM) (3) studies revealed an inverse relationship between serum concentrations of high-density lipoprotein (HDL) and the risk of cardiovascular disease (CVD), independently of the levels of low-density lipoprotein cholesterol (LDL-C). However, in recent decades it has been increasingly questioned whether approaches to improve HDL-related atheroprotective system function will actually reduce the risk of atherosclerosis. The CANHEART (4) study showed that high levels of HDL cholesterol (HDL-C) did not reduce mortality from CVD, indicating that high HDL-C is not necessarily associated with cardioprotection. Also, a genetic study showed that three functional variants of hepatic lipase associated with a modest increase in HDL-C levels did not reduce cardiovascular risk (5). On the other hand, a 29.3% reduction in HDL-C levels due to functional mutations in the ATP Binding Cassette-A1 (ABCA1) transporter did not adversely affect cardiovascular risk (6). Mendelian randomization studies demonstrated a lack of causal link between low HDL-C and the development of atherosclerosis (7), that is, low HDL-C levels are strongly associated with an increased risk of myocardial infarction, but when these levels are genetically determined, an association does not exist (7). However, studies of HDL functionality showed an even more evident association with cardiovascular risk than HDL-C levels (8), indicating that HDL functionality plays a more relevant role in atheroprotection than its circulating levels.

Very low HDL-C is seen in clinical conditions that differ from each other in terms of their potential for the development of atherosclerotic disease. Individuals afflicted with single-gene diseases are at high risk of premature atherosclerosis (9). On the other hand, less severe mutations in genes related to intravascular metabolism or HDL synthesis (10) do not result in an increased risk of atherosclerotic disease (11). In contrast, low plasma HDL-C can also be seen in insulin resistance and subsequent atherogenic dyslipidemia, characterized by low HDL-C levels, high triglyceride levels, and an increased proportion of small, dense lipoproteins both HDL and low-density lipoprotein (LDL) (12). In this review, we will detail the clinical conditions that occur with low levels of HDL-C.

### HDL metabolism

HDL particles are present in the circulation in different sizes (7-12 nm) and densities (1,063-1,21 g/mL) and represent the HDL pool in the course of its maturation after hepatic and intestinal production of apolipoprotein (apo)A-I, which is functionally and structurally the most important protein of HDL. Pre-β, discoid, small and lipid-poor HDL captures free cholesterol after binding between apoA-I and the ABCA1 receptor of peripheral cells, allowing reverse cholesterol transport (13).

At the same time, HDL particles are also enriched with phospholipids derived from other lipoproteins, especially very low-density lipoprotein (VLDL), by phospholipid transfer protein (PLTP). For the formation of HDL particles with a lipid and hydrophilic core, one of the enzymes associated with HDL, the enzyme lecithin-cholesterol acyltransferase (LCAT), catalyzes the transfer of fatty acids from phospholipids to cholesterol, esterifying it and promoting the formation of HDL particles rich in cholesterol esters. Reverse cholesterol transport is completed when HDL is taken up by hepatic Scavenger Receptor-B1 (SR-B1) receptors, initiating the process of excretion through the bile. On the other hand, cholesteryl esters can also be transferred to lipoproteins that contain apoB by the action of Cholesteryl Ester Transfer Protein (CETP), through exchange for triglycerides (14).

As a carrier of several proteins, including acute phase enzymes, and a small amount of non-polar lipids, HDL is found in the circulation with different phenotypes and biological properties. While some of these actions (such as antioxidant activity) are intensely exerted by immature forms of HDL (HDL3), reverse cholesterol transport and anti-inflammatory action on the endothelium appear to require more mature forms of HDL (HDL2) (15). This broad set of actions is the result of several components of HDL, such as apoA-I, apoA-II, apoJ, as well as other proteins, enzymes, phospholipids and even probably microRNAs that are transported by HDL particles (16,17).

### Monogenic dyslipidemias

Extremely low serum HDL-C levels (<30-35 mg/dL) in the absence of secondary causes occur in 1% of the general population (18) and correspond to rare and underdiagnosed genetic syndromes caused by mutations in genes that regulate HDL production or catabolism. According to population studies, 18.7% of individuals with very low HDL-C carry rare genetic variants of great effect and 19.3% carry common low-effect variants (19). Thus, the genetic basis of very low HDL-C disease is often polygenic.

### Tangier disease

**Figure 1:**
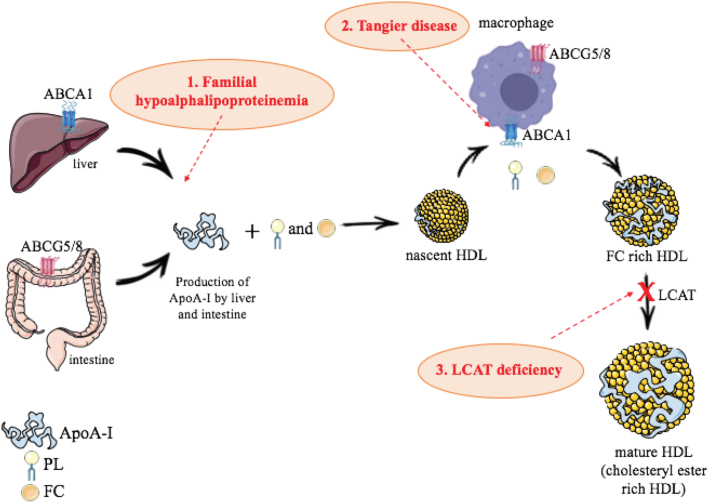
HDL regulation and monogenic disorders with low levels of HDL-C. Black arrows show HDL metabolism and red arrows where monogenic disorders occur in the three conditions reported in this review. **1.** The subjects with familial hypoalphalipoproteinemia have been reported to have either decreased HDL production or increased HDL ApoA-I catabolism. **2.** The disorder results from homozygous mutations in the ABCA1 transport protein, which mediates the efflux of cellular cholesterol to the HDL particle in plasma for transport to the liver. **3.** Both FLD and FED are caused by the mutations in the LCAT gene. Both disorder lead to a marked reduction in plasma HDL-C. ApoA-I: apolipoprotein A-I; PL: phospholipids; FC: free cholesterol; PLTP: Phospholipid Transfer Protein; LPL: lipoprotein lipase; ABCA1: ATP-binding cassette transporter A1; LCAT: Lecithin-cholesterol acyltransferase; ABCG5/8: ATP-binding cassette, subfamily G, member 5 and member 8.

The ABCA1 gene resides on chromosome 9q22-q31, contains 50 exons and encodes a long membrane protein of 2,261 amino acids. It consists of two transmembrane domains, each formed by six alpha helices and two intracellular nucleotide-binding domains (20). Tangier disease is a rare monogenic autosomal recessive disorder that occurs due to mutations in both alleles of the ABCA1 gene, both exonic (21) and intronic in ABCA1 causing aberrant splicing of mRNA (22). Overall, as changes in the ABCA1 gene negatively influence cellular cholesterol uptake, the concentration of HDL-C decreases more sharply and the incidence of coronary artery disease (CAD) increases. Fibroblasts are very deficient in the export of cholesterol and, as a result, complete particles of HDL are not formed (23). Therefore, ABCA1 deficiency leads to intracellular accumulation of cholesteryl esters, precluding the conversion of the lipid-poor apoA-I particles into pre-β HDL (24) (Figure 1).

### Clinical assessment

Characterized by the almost complete absence of HDL-C (always less than 5 mg/dL), homozygous individuals show a marked increase in apoA-I catabolism, with plasma residence time of about 0.5 days. On the other hand, heterozygous individuals show increased clearance with plasma residence time of about 2 days (25). Also, heterozygotes may show a reduction of approximately 50% in ABCA1-mediated cellular cholesterol efflux (24). There is an accumulation of cholesterol esters in the reticuloendothelial system and adipose tissue, in addition to yellow-orange hyperplastic tonsils, which, together with very low levels of HDL-C, are considered indicative of the disease. Individuals have elevated TG and a reduction of up to 50% in LDL-C concentration.

There are controversies in the literature as to whether individuals who are homozygous for Tangiers have an increased risk of developing premature CVD. An analysis involving 185 cases showed that 25% had CVD but among individuals over 40 years of age this prevalence reached 52% compared to 11% in age- and gender-matched controls (25). This suggests that these individuals do not develop early CVD because LDL-C levels are about 50% of normal. Furthermore, this report suggested the presence of two distinct phenotypic groups: (i) patients with marked hepatosplenomegaly, anemia, low levels of non-HDL-C (<70 mg/dL) and absence of premature CAD; and (ii) patients without hepatosplenomegaly or severe anemia, normal or near-normal non-HDL-C levels (>70 mg/dL) and premature CAD (25). The variability in CVD risk in homozygotes may be, in part, explained by non-HDL-C levels.

In many cases, peripheral neuropathy is absent or only detectable by electrophysiological investigation of nerve conductance velocity. On the other hand, symptomatic individuals present: (i) a multifocal demyelinating form that is mononeuropathic or asymmetric polyneuropathic and affects the motor and sensory nerves of the limbs or head or (ii) a syndrome similar to syringomyelia, being progressive and often debilitating (20).

### Clinical management

ABCA1 molecular gene sequencing is the gold standard for diagnosing Tangier disease. However, two tests can help make the diagnosis more likely: two-dimensional non-denaturing electrophoresis and anti-apoA-I immunoblotting help distinguish part of HDL that has alpha electrophoretic mobility (α-HDL) from a quantitatively smaller proportion that has pre-electrophoretic mobility (pre β1-HDL) (26).

Other complementary tests may be performed: nerve conduction studies and electroneuromyography to determine the presence of peripheral neuropathy; ophthalmologic evaluation to identify any corneal opacities; abdominal ultrasound to assess hepatosplenomegaly; carotid Doppler ultrasound to identify the thickness of the intima and media layers of the carotid arteries (cIMT) and plaques (27); and echocardiogram to assess coronary atherosclerosis.

### LCAT deficiency

LCAT deficiency is a very rare autosomal recessive disorder. The majority of described genetic variations are loss-of-function mutations leading to proteins with total or partial lack of LCAT activity (28). Genetic LCAT deficiency is associated with the development of two syndromes: (i) familial LCAT deficiency (FLD) and (ii) fish-eye disease (FED). Among the described mutations, 53 are associated with an FLD phenotype and 19 mutations lead to a FED phenotype (28). FLD is characterized by mutations that result in the absence or complete inactivity of the LCAT enzyme and present the disease in its severe form, while FED is the result of mutations that inhibit the ability of LCAT to esterify cholesterol to HDL, but do not affect the ability of esterifying cholesterol to lipoproteins that have apoB, so patients are relatively less symptomatic (29) (Figure 1).

In a case series of a Canadian family with LCAT deficiency, no cardiovascular events or deaths were reported for 25 years after the initial diagnosis. In the two homozygous individuals, cIMT was above the 75th percentile expected for age and sex. However, the abnormalities were much more pronounced in the heterozygous individuals, four of whom had detectable plaques, indicating that heterozygosity may be associated with an atherogenic lipid profile and vascular abnormalities (30). Notably, homozygous carriers have low plasma concentration of LDL-C, which would explain protection against atherogenesis.

A more extensive study evaluated cIMT in 40 carriers of LCAT gene mutations and in 80 healthy controls and found no significant difference between mutation carriers (31). Divergent results between studies remain unclear about whether low LCAT activity, genetically determined, is associated with increased preclinical atherosclerosis. LCAT, by itself, is not the only regulator of reverse cholesterol transport pathways (32). Both overexpression and LCAT deficiency showed reverse cholesterol transport from macrophages preserved in an animal model (33). Furthermore, plasma from LCAT-deficient individuals has the same ability to reduce the cholesterol content of macrophages compared to plasma from control individuals (34). Thus, it remains questionable whether the elevation of LCAT activity is a promising therapeutic strategy to reduce cardiovascular risk.

### Clinical assessment

Individuals affected by FLD develop corneal opacification, anemia and notable proteinuria (35). Also, the patients develop early and progressive chronic kidney disease leading to early end-stage renal disease, which is the main cause of morbidity and mortality in this population (36). The reason for the deterioration of renal function remains unknown. Heterogeneous glomerular pathology implies several possible mechanisms, including abnormal LDL trapping and C3 complement deposition (37). The presence of anemia may occur in association with the disease and is attributed to an increase in the fragility of erythrocytes due to the abnormal lipid composition of their cell membrane. On the other hand, individuals with FED clinically have corneal opacification but are spared anemia and kidney disease and are therefore considered to have a milder form of LCAT deficiency (38).

Corneal opacification, the characteristic physical finding in LCAT deficiency state, is more severe than the accumulation of cholesterol in the cornea that can be sporadically seen in apoA-I deficiency and Tangier disease. Patients with FLD and FED slowly develop progressive corneal opacification accompanied by a white or gray ring at the corneal margin similar to arcus senilis (20). Surprisingly, vision remains intact in most cases.

Prevalence of CAD is higher in FED than in FLD, and this is supported by significantly lower LDL-C in carriers of FLD mutations compared with carriers of FED mutations (39); however, there are case reports of mutations in LCAT that present low or normal plasma levels of HDL-C without premature CAD (34). Assessment of cIMT, a surrogate imaging measure of cardiovascular risk, also suggests that there is no significant worsening of the vascular phenotype among individuals that are homozygous for LCAT mutations.

### Clinical management

Initial clinical suspicion is raised based on corneal opacification. The diagnosis of these disorders can be performed through the quantification of LCAT. Definitive diagnosis requires molecular genetic testing of the LCAT gene and functional analysis of the gene product.

Patients with FLD and FED have not only very low levels of HDL-C (<10 mg/dL), but also elevations in VLDL enriched with free cholesterol (25). In two-dimensional gel electrophoresis there is the presence of pre-β HDL and α4-HDL and the absence of mature α-HDL (28). ApoA-I levels are usually between 20-30 mg/dL and LDL-C concentration is often low. In addition, they have a large, heterogeneous LDL phenotype enriched in free cholesterol, phospholipids and TG, with a very low cholesterol ester content. These particles are also low in apoB and enriched in apoC. The lipid profile is also characterized by the presence of lipoprotein X (Lp-X). Lp-X is a particle that has low protein, esterified cholesterol and TG content, and high free cholesterol and phospholipid content (40). Experimental studies demonstrate that Lp-X is nephrotoxic, and there is an association with FLD-related kidney disease, showing distinctive lipid deposits in glomeruli in histology, but not with FED (41).

### Familial hypoalphalipoproteinemia

Familial hypoalphalipoproteinemia is a very rare autosomal dominant disorder characterized by a heterogeneous group of mutations that cause apoA-I deficiency resulting from a biallelic mutation in the *APOA1* gene, located on chromosome 11q23.3, which contains four coding regions and is clustered with the apoC-III and apoC-IV genes (42). Functionally significant mutations have also been described, including gene disruptions, frame changes, nonsense mutations, chromosomal aberrations or deletions, as well as *APOA1/APOA3/APOA4* gene cluster inversion associated with decreased HDL-C levels (43-45). ApoA-I variants are generally heterozygous premature terminations, frameshifts, or amino acid substitutions in the 243 amino acid sequence of apoA-I (25) (Figure 1).

### Clinical assessment

Although extremely low HDL-C can be detected in any patient from birth, the age of onset of symptoms and the clinical presentation vary widely. Homozygous or compound heterozygous carriers have two different clinical features: (i) xanthomas or (ii) corneal opacities. Cutaneous xanthomas (tuber-eruptive, tendinous, palmar or planar) have been described in adult patients who are homozygous or compound heterozygous carrying null alleles and therefore do not have apoA-I in their plasma. They often have premature CAD and carotid atherosclerosis (46).

Heterozygous variants of apoA-I that cause low HDL-C and decreased LCAT activation are not associated with premature CVD. However, apoA-I variants associated with low HDL-C and normal LCAT activity have been associated with premature CVD (25). Heterozygous carriers of apoA-I variants do not show specific clinical symptoms. An important exception is some structural variants of apoA-I with amino-terminus amino acid substitutions, detected in patients with familial amyloidosis (47-49). These amyloidogenic mutations lead to the accumulation of amyloid containing amino-terminus apoA-I fragments (9-11 kd N terminal fragments (25) in the liver, intestine, kidney, heart, peripheral nerves and skin (47-49). ApoA-I deficiency can also manifest with sensorineural signs such as cerebellar ataxia, sensorineural hearing loss, and proliferative retinopathy.

In addition, some apoA-I variants such as apoA-I (L178P) or apoA-I (L159P) were associated with increased risk of premature CAD or increased progression of cIMT (50,51), while others did not show this association, or even proved to reduce cardiovascular risk remarkably, such as apoA-I Milano (52).

### Clinical management

Homozygous carriers have undetectable apoA-I (less than 5 mg/dL) and HDL-C lower than 10 mg/dL. On the other hand, heterozygous carriers generally have HDL-C levels that are often below the fifth percentile or, at least, below the cardiovascular risk threshold level of 40 mg/dL for men and 50 mg/dL for women. As would be expected, apoA-I levels are also frequently below the fifth percentile (<105 mg/dL in men and <110 mg/dL in women) (53).

In a patient with xanthomas, histological examination of the skin lesions reveals numerous foam cells. Molecular diagnosis can be performed by the technique of two-dimensional gel electrophoresis observing the absence or reduction of apoA-I. DNA analysis can also be useful for an accurate diagnosis, performed by sequencing the *APOA1* gene and demonstrating a functionally relevant mutation. Many structural variants of apoA-I can be detected by isoelectric focusing and anti-apoA-I immunoblotting (53).

### Polygenic dyslipidemias

Recent studies indicate that the genetic basis of extreme concentrations of HDL-C identified clinically is often polygenic, having previously been considered an archetypal “monogenic” disorder. As mentioned above, about 18.7% of individuals with very low HDL-C are heterozygous for rare mutations of great effect and 19.3% of the cases of very low HDL-C show an accumulation of common mutations (19). In addition, some patients with polygenic dyslipidemia may also have mixed dyslipidemias (low HDL-C and elevations of TG) associated with a different clinical condition such as metabolic syndrome or obesity. Identification of single nucleotide polymorphisms (SNP) that have modest effects on HDL-C plasma have been used to confirm or refute the role of HDL in the development of atherosclerotic disease and as a tool to identify individuals with polygenic dyslipidemias. A classic Mendelian randomization study demonstrated that polymorphism in the lipase gene endothelial cell and a genetic score of 14 common SNP that specifically increased HDL-C were not associated with risk of acute myocardial infarction, suggesting that some genetic mechanisms that increase or decrease HDL-C do not reduce cardiovascular risk (8). Another study that used SNP of the target genes as an instrument demonstrated that not all metabolic alterations that modify serum HDL-C levels influence cardiovascular risk (54).

Consistently, studies that used randomization Mendelian research revealed that while the increase of polygenic origin in the concentration of lipoproteins associated with apoB is associated with increased risk of CVD, no association was found with SNP-mediated variation in lipoproteins associated with apoA-I (55,56). Although these findings may suggest the absence of a causal relationship, plasma levels of HDL-C or apoA-I do not represent reliable instrumental variables, considering that the increase in the circulating concentration of HDL-C or apoA-I does not always equate with a change of roles of the HDL particle or its interaction with cells, i.e., in the HDL system.

### Secondary dyslipidemias with low HDL-C

#### Type 2 diabetes mellitus, insulin resistance, and obesity

Some dyslipidemias are not explained by mutations but are related to epigenetic mechanisms that may be influenced by drug interventions, lifestyle and environmental factors. Reduced levels of HDL-C are often present in resistance to insulin, obesity and type 2 diabetes mellitus (T2DM) and are associated with hypertension and dyslipidemia, factors that can lead to the early development of CAD. In fact, dyslipidemia in T2DM is observed in 60%-70% of patients and is characterized by high levels of TG and decrease in HDL-C, with this reduction being an independent factor not only for the development of CVD, but also for the manifestation of T2DM (57). Also, insulin resistance is known to be associated with lower circulating HDL-C (58). These changes together with the presence of small and dense LDL particles contribute to accelerating atherogenesis. In this clinical condition, one of the mechanisms that promote the reduction of HDL-C is elevation of TG-rich lipoprotein levels that, through CETP, transfer TG to HDL in exchange for cholesterol esters (59).

Subsequent removal of this excess HDL-TG by hepatic lipase results in smaller and denser HDL particles. HDL size of person with diabetes is altered, with loss of large and very large HDL2 and gain of small HDL3 (60). The same mechanism promotes the formation of small and dense LDL (61). In this group of individuals, sub analyses of studies with fibrates and studies with an eicosapentaenoic acid derivative, icosapent ethyl, were associated with a reduction in cardiovascular risk (62). It is not possible, however, to attribute the benefit of this therapy, even if partially, to the increase in HDL-C. So, although the isolated role of HDL is unclear in atherogenic dyslipidemia, its diagnosis remains a prognostic marker in therapies.

#### Use of anabolic androgenic steroids

Anabolic androgenic steroids (AAS) are synthetic derivatives of testosterone and are widely used by athletes to improve their physical performance. Observational studies have shown that the use of AAS is associated with adverse effects, including significant decrease in HDL-C levels and increase in LDL-C (63). The mechanisms by which AAS affect HDL-C concentrations are not completely elucidated. Until now, it is known that AAS stimulate hepatic lipase enzyme activity in order to favor the catabolism of HDL and inhibit the biosynthesis of apoA-I (64). In contrast to cross-sectional and prospective observational studies, one study showed hypogonadal patients, characterized by very low testosterone, with a lipoprotein profile within the normal range, suggesting a relationship with the absence of clearly defined obesity. However, there was a marked reduction in HDL cholesterol efflux capacity and an increase in serum cholesterol carrying capacity (65). Also, there was a decrease in HDL-C concentrations during transgender hormone therapy. HDL cholesterol efflux capacity decreased during hormone therapy with specific reduction in ABCA1, contributing to an increased risk of CVD (66). Cholesterol efflux capacity is a metric of HDL functionality that quantifies the ability of an individual’s HDL to extract cholesterol from macrophages. It has been shown to be a better predictor of atherosclerotic burden than HDL-C levels alone (67).

#### Infectious diseases

During infection, significant changes occur in lipid metabolism and lipoprotein composition. Lipoprotein concentrations rapidly change and can be reduced to 50% of recovery concentrations. Also, the levels of circulating HDL-C and LDL-C decrease, while the levels of TG and VLDL-C increase (68), so it is thought that HDL-C may be a negative marker for systemic or local inflammations. More importantly, endotoxemia modulates HDL composition and size: phospholipids and apoA-I are reduced, while serum amyloid A (SAA) and secretory phospholipase A2 (sPLA2) increase dramatically. Although the number of total HDL does not change, a significant reduction is observed in the number of small and medium-sized particles (69).

HDL can bind to and neutralize bacterial lipopolysaccharide gram-negative and gram-positive bacterial lipoteichoic acid, favoring the debugging of these products (70). Interestingly, HDLs also play a role in fighting infection parasites, and a specific component of HDL, the apolipoprotein L-1 (apoL-1), confers innate immunity against *Trypanosoma cruzi* by favoring the lysosomal swelling that kills the parasite (68). At the same time that HDL-C undergoes a reduction in infectious diseases, low concentrations of HDL-C are also associated with an increased risk of acquiring infections, and prospective cohort studies observed a U-shaped relationship between HDL levels and risk of infectious disease (71). In addition, a Mendelian randomization study demonstrated that, genetically, certain HDL-C levels have a significant influence on risk of hospitalization for infectious diseases (72).

### Therapeutic approaches

Mortality from CVD remains the leading cause of death in industrialized countries, and although the use of lipid-lowering therapies focused on LDL-C reduction has made a remarkable contribution to its control, there is still a substantial residual risk of CVD mortality. The observation of this residual risk has driven the search for new therapeutic targets, including conventional and new pharmacological therapies that are still under development. The great challenge lies in improving the functionality of the HDL system to favor the antiatherothrombotic effect. In the following paragraphs, we will briefly comment on some tested or researched therapies for this purpose. A table summarizing the main clinical trials and their outcomes is found at the end of this review (Table 1).

**Table 1:** Major clinical trials conducted involving therapies to increase plasma HD levels

Drug class	Study	Treatment	Comparator	Changes in HDL-C	Outcomes
iCETP	ILLUMINATE	Torcetrapibe plus Atorvastatin	Atorvastatin	Increase HDL-C by 72%	Increase in CV events and death
	REVEAL	Anacetrapib	Placebo Anacetrapib	Increase HDL-C by 104%	Lower incidence of major coronary events
	Dal-OUTCOMES	Dalcetrapibe	Placebo	Increase HDL-C by 30%	Treatment had no significant effect on major CV outcomes
	ACCELERATE	Evacetrapibe	Placebo	Increase HDL-C by 133%	Treatment did not result in a lower risk of death from CV causes
Fibrates	FIELD	Fenofibrate	Placebo	Increase the levels of HDL-C of 5%	The results showed a non- significant 11% relative reduction in the primary outcome of first MI or CHD death
	ACCORD	Fenofibrate	Placebo	Increase the levels of HDL-C of 7.9%	The results showed that the combination of fenofibrate and simvastatin did not reduce the rate of fatal CV events
	PROMINENT	Pemafibrate	Placebo	-	Study data will be presented as soon as possible at a future conference
Niacin	AIM-HIGH	Extended-Release Niacin plus Statin	Placebo plus Statin	HDL-C level increased by 25%	There was no incremental clinical benefit from the addition of niacin to statin therapy
	HPS2 - THRIVE	Niacin - Laropiprant	Placebo	HDL-C level increased by 35%	Treatment increased the risk of serious adverse events
Recombinant HDL	ERASE	CSL-111	Placebo	Not evaluated	Short-term infusions of reconstituted HDL resulted in no significant reductions in percentage change in atheroma volume
	AEGISII Phase 3 Study	CSL-112	Placebo	-	CSL-112 has been well tolerated and we will have more information soon
	MODE	CER-001	-	No significant changes were observed in HDL-C in patients with genetically confirmed homozygous or compound heterozygous FH	Reduction in the volume and area of plaque on the vascular wall carotid
	CHI-SQUARE	CER-001	Placebo	-	CER-001 infusions did not reduce coronary atherosclerosis on IVUS and QCA when compared with placebo
	MILANO-PILOT	MDCO-216	Placebo	MDCO-216–treated patients demonstrated reductions in HDL-C of 8%	Failed to show plate volume regression
Recombinant LCAT	Clinical Trials NCT01554800	ACP-501	-	Increase the levels of HDL-C by 44%	ACP-501 had an acceptable safety profile
	Clinical Trials NCT02601560	EDI6012	Placebo	Dose‐dependent increases in HDL‐C	MEDI6012 demonstrated an acceptable safety profile
ApoA-I transcriptional supraregulators	ASSURE	RVX-208	Placebo	HDL-C increased by 11.1%	There were no incremental reductions in other atherogenic lipid parameters or high-sensitivity CRP with RVX-208

CV: cardiovascular; MI: myocardial infarction; CHD: coronary heart disease; IVUS: intravascular ultrasonography; QCA: quantitative coronary angiography; CRP: C-reactive protein.

#### Statins

Statin therapy has been shown to increase the level of plasma HDL-C and the mechanism most likely involves reduced transfer of cholesteryl ester from HDL to VLDL but other factors such as hepatic lipase and other statin-induced effects may also contribute (73). Due to the inhibition in HMG-CoA reductase and suppression of Rho activity, statins can stimulate the synthesis of apoA-I in a dose-dependent manner and increase ABCA1 expression (74,75). In parallel, statins inhibit CETP synthesis and bioavailability of lipoproteins rich in TG, which may contribute to the increase of HDL-C (76).

Data from randomized clinical trials observed a two-fold increase in HDL-C levels compared to apoA-I levels (77). Furthermore, the percentage increases in HDL-C levels were greater in those individuals who had lower baseline plasma HDL-C levels (78).

These data show that statins alter HDL to a more cholesterol-rich form, characteristic of healthy populations of low cardiovascular risk (78). According to the STELLAR clinical study, among a group of five statins, the ability to increase HDL-C was greatest with rosuvastatin (9.2%), followed by simvastatin (6.8%), atorvastatin (5.7%) and, finally, pravastatin (5.6%) (77). Data from large studies of morbidity and mortality suggested that the effects induced by statins in HDL-C and apoA-I were sustained over time, offering an interesting advantage in relation to HDL infusion therapies, for example, whose effect is short-term (79). The clinical impact of increased HDL-C by statin treatment is independent of its effects on LDL-C levels, TG-rich lipoproteins, or other side effects.

#### CETP inhibitors

CETP inhibitors were developed with the aim of blocking or interfering with CETP activity. They can be characterized into CETP inhibitors (torcetrapib, anacetrapib and evacetrapib) and CETP modulators (dalcetrapib) according to their chemical structure (80). The main function of CETP is the transfer of cholesteryl esters and TG between plasma lipoprotein particles. CETP inhibitors target a part of this mechanism by blocking the transfer of cholesteryl esters (81).

CETP inhibitors increase HDL-C levels significantly (40% to 160%) due to the decrease in the transfer of cholesterol from HDL particles to rich lipoproteins in TG (82). However, three clinical trials failed to demonstrate any cardiovascular benefits with treatment with these inhibitors (83-85). The REVEAL study (86) showed that cardiovascular events occurred less frequently in patients with atherosclerotic vascular disease treated with anacetrapib after 4.1 years of follow-up. In this last trial there was an 18% reduction in non-HDL cholesterol and a 104% increase in HDL-C with anacetrapib. A new CETP inhibitor, obicetrapib, reduced LDL-C and apoB levels by 45.3% and 33.7%, respectively. While HDL-C levels increased by 179.1% and ApoA-I by up to 63.4% (87).

A Mendelian 2 × 2 factorial randomization study including 425,354 UK Biobank participants showed an additive association of a genetically reduced combined concentration of CETP and PCSK9 for lipid levels and risk of CAD. Suggesting that the joint inhibition of CETP and PCSK9 has additive effects on lipid concentrations (88). However, the double-blind dal-GenE study in patients with acute coronary syndrome of 1-3 months and the AA genotype in the rs1967309 variant in the ADCY9 gene showed that dalcetrapib did not significantly reduce the risk of ischemic cardiovascular events at the end of the study (89). A prospective, multicenter, cohort study verified the association between very high HDL-C levels and mortality in patients with CAD and the association of HDL-C genotypes with high HDL-C outcomes. The authors found a U-shaped association with higher risk in those with low and high levels of HDL-C compared with those with medium values (90). Paradoxically, the study suggests that very high levels of HDL-C are associated with a higher risk of mortality in individuals with CAD.

#### Fibrates

Fibrates are agonist drugs at peroxisome proliferator-activated receptor-α (PPAR-α) nuclear receptors, responsible for regulating the transcription of the LPL, ApoC-III and ApoA-I genes, leading to the clearance of TG-rich lipoproteins (91). Fibrates promote stimulation of HDL production by inducing hepatic synthesis of ApoA-I and ApoA-II and reducing VLDL production due to the reduction of free fatty acids in the liver, in addition to inhibiting the exchange of cholesterol and TG between HDL and VLDL (92). By inducing elevation of HDL-C levels, reduction of TG-rich lipoproteins, and a shift in the phenotype from dense LDL to receptor-active, floating LDL, fibrates act to attenuate the atherosclerotic burden in atherogenic dyslipidemia (91).

A reduction in cardiovascular events was observed in patients with low HDL-C after treatment with gemfibrozil without the addition of statins (93,94). Although bezafibrate has been more effective than gemfibrozil in raising HDL-C levels, its use in secondary prevention has not proven to be beneficial in reducing cardiovascular events in patients with low HDL-C (95). In patients with T2DM, fenofibrate therapy also did not show cardiovascular benefits even in those with concomitant use of statins (96,97). A new category of PPAR-α selective modulators that has been studied, pemafibrate, significantly reduces TG, apoC-III, and cholesterol remnants, in addition to increasing HDL-C. The PROMINENT study aims to evaluate the effect of pemafibrate on cardiovascular events in approximately 10,000 persons with diabetes with moderately elevated TG and low HDL-C (98). However, the Kowa Research Institute announces the decision not to continue the Phase 3 PROMINENT study. Based on a review of a planned interim analysis, they concluded that the primary endpoint would likely not be met. Full study data will be presented as soon as possible at a future conference.

#### Niacin

Through its GPR109A receptor (G-protein-coupled receptor) present in adipocytes, niacin acts by inhibiting the mobilization of free fatty acids, thus decreasing the supply of substrate for hepatic synthesis. It is possible that niacin increases HDL-C by its ability to inhibit hepatic lipase, decrease the catabolic rate of HDL-C, and decrease catabolic HDL-C receptors. These various effects result in higher levels and larger HDL-C molecules and more efficient reverse cholesterol transport (99). In hepatocytes, niacin inhibits the activity of diacylglycerol O-acyltransferase 2 (DGAT-2), decreasing the hepatic synthesis of TG and VLDL (100), in addition to promoting selective inhibition of ApoA-I uptake. Among the therapies that increase the serum concentration of HDL-C, niacin has the greatest effect, with an increase of 15 to 40% (101), preferentially raising the HDL2 subfraction. A meta-analysis showed that niacin was able to increase HDL-C levels by 23%, in addition to reducing TG levels by 40% and LDL-C levels by 20% (102). Despite being very effective in increasing HDL-C there is no evidence that the addition of niacin to therapy with statins results in cardiovascular benefit. Randomized clinical trials tested this hypothesis and failed to demonstrate any benefit from adding niacin to therapy with statins (103,104).

#### Therapies based on recombinant HDL

HDL infusion therapies induce a rapid, dose-proportional, time-dependent elevation in apoA-I and pre-βHDL particles. These effects were seen for recombinant HDL (CSL-111 and CSL-112), where plasma concentrations of apoA-I increased 2-fold and pre-βHDL increased >30-fold (79,105). Despite this, the ERASE trial (106) showed that CSL-111 infusions did not result in significant reductions in atheroma volume or nominal change in plaque volume but did significantly impact the plaque characterization index on intravascular ultrasound. The AEGISII Phase 3 Study (expected completion 2022) (NCT03473223) assesses the efficacy and safety of CSL-112 in reducing larger cardiovascular events and will provide more information about the effect of this treatment in the progression of CVD.

The CER-001, an engineered negatively charged lipoprotein particle that contains recombinant human apoA-I (107), induced dose-dependent increases in plasma HDL-C of up to 7-fold (108). In the MODE study (109), CER-001 was administered in serial infusions in 23 patients with homozygous familial hypercholesterolemia suggesting reduced mean carotid wall thickness. In the CHI-SQUARE study (110), however, its administration did not reduce the coronary atherosclerotic burden. Interestingly, CER-001 appears to have a therapeutic target in LCAT deficiency, as shown in LCAT-/- mice treated with CER-001, there was improvement in dyslipidemia and renal function (111). They also verified the ability of CER-001 to remove cholesterol, reducing the deposition burden on the kidney in a patient with FLD. The beneficial effect is mediated by a dual action of CER-001, which directly effuses cholesterol from podocytes, but also induces the normalization of plasma lipoproteins (112). This beneficial effect occurs earlier than predictable and may offer a new form of treatment for this serious condition (113).

In a phase 2 clinical study, administration of ETC-216, recombinant HDL with apoA-I Milano, produced significant regression in atherosclerotic plaque volume in subjects with acute coronary syndrome, measured by intravascular ultrasound (114) suggesting that infusion in the first weeks after an acute event would stimulate the reverse cholesterol transport. However, serious side effects prevented further evaluation of this formulation and development of this formulation stopped. MDCO-216, tested in a multicenter trial, showed that after five weekly infusions, the percentage volume of the atheroma in the diseased segment did not differ significantly between patients with coronary heart disease documented by angiogram and the control group, ending its development (115).

#### Recombinant LCAT (rhLCAT)

Most data from preclinical studies suggest that increasing the amount of LCAT stimulates reverse cholesterol transport and reduces atherosclerosis. These data are based on studies that observed a reduction in the progression of atherosclerosis in mice with increased expression of LCAT (116), in addition to the increase in atherosclerotic lesions in mice with enzyme deficiency (117). In this context, a phase I clinical trial demonstrated that a single infusion injection of rhLCAT (ACP-501) had an acceptable safety profile and favorably altered HDL metabolism, showing a dose-proportional increase in HDL-C, thus supporting the investigation of this therapy in individuals with atherosclerotic disease and LCAT deficiency (118). Next, in the FLD patient study, intravenous drip administration of ACP-501 improved the abnormal distribution of HDL subfractions (119). MEDI6012 is a more active rhLCAT that resulted in increased HDL function in a dose-related manner and also promoted endothelial protection (120). Furthermore, LCAT overexpression improved LDL receptor-mediated reverse cholesterol transport, leading to regression of atherosclerosis, suggesting synergistic effects of MEDI6012 with statins (121).

#### ApoA-I mimetic peptides

ApoA-I is the main apolipoprotein present in HDL particles. Although it is composed of 243 amino acids arranged in 10 amphipathic helices, there has been interest in using short apoA-I peptides containing at least one amphipathic helix to mimic the functions of native apoA-I. The first apoA-I mimetic peptide was synthesized comprising 18 amino acids (18A). Subsequently, several modifications to 18A were made to create peptides that more closely mimic apoA-I in its antiatherogenic functions (122). Then the addition of an acetyl group and an amide group promoted stability and the lipid binding properties were improved, the peptide was named 2F. Although the 2F peptide mimicked many of the lipid-binding properties of apoA-I, it failed to alter the lesions in a mouse model of atherosclerosis (123). Subsequent, the administration of peptide 5F by injection significantly inhibited lesion formation in mice fed an atherogenic diet without significantly altering lipoprotein profiles (124). Another apoA-I mimetic peptide synthesized from D-amino acids (D-4F) to LDL receptor null mice on a Western diet made HDL anti-inflammatory and reduced lesions by 79% without altering HDL-C levels (125). Recently, P12 showed efficient binding with cytomembrane phospholipids, cholesterol and HDL, and showed the ability to reverse cholesterol transport and treat atherosclerosis (126). With its phospholipid affinity, P12 facilitated cholesterol transport through the ABCA1. Also, promoted the function of HDL by remodeling α‐HDL into preβ‐HDL which can more efficiently transport cholesterol in the atherosclerotic plaques to the liver and remove excess cholesterol from the arteries (126). The use of peptides that mimic HDL-associated apolipoproteins may prove to be a successful strategy in the treatment of vascular diseases.

#### ApoA-I transcriptional supraregulators

RVX-208 is a therapeutic agent that selectively induces hepatic de novo synthesis of apoA-I and thereby increases HDL levels accompanied by a change in the pattern of HDL distribution. In monkeys, it primarily increased the levels of pre-β1 and α-1 HDL particles, representing the lipid-poor cholesterol efflux receptors and the larger lipid-rich HDL particles, respectively (127). Treatment with RVX-208 may cause, through de novo synthesis of apoA-I and/or decrease in LCAT activity, favorable changes in HDL distribution. In humans, a 42% and 11% increase in pre-β1 HDL levels and ABCA1-mediated cholesterol efflux, respectively, occurred after only 7 days of RVX-208 therapy (127). However, the ASSURE study showed modest increases in apoA-I and HDL-C with RVX-208, these changes did not differ from the placebo group. These data demonstrate that treatment with RVX-208 did not result in any measurable incremental benefit in plaque regression for patients with CAD and low HDL-C levels (128).

#### microRNA antagonists

Studies have identified miR-33a/b as essential regulators of lipid metabolism, regulating HDL-C levels and the multi-step reverse cholesterol transport process, therefore, the therapeutic potential of miR-33 to treat CVD is promising. The antagonism of miR-33 significantly increased plasma levels of HDL-C directed macrophage polarization to an M2-like phenotype and improved atherosclerosis regression (129). However, two independent groups showed a beneficial effect of miR-33 inhibition in attenuating atherosclerosis progression (130,131), while another group did not observe any improvement in atherogenesis under similar conditions (132). Interestingly, all three studies demonstrated that miR-33 antagonism in hypercholesterolemic mice does not increase plasma levels of HDL-C. These findings suggest that miR-33 inhibition may promote atheroprotection through mechanisms independent of the increase in circulating HDL-C.

#### Antisense oligonucleotides for CETP and ApoC-III

One study compared an antisense oligonucleotides (ASO) inhibitor of CETP with the inhibitor of anacetrapib in hyperlipidemic mice and both therapies resulted in a decrease in total plasma cholesterol, decrease in CETP activity and increase in levels of HDL-C (133). ASO against ApoC-III decreases its production and, consequently, lipolysis enhancement of TG-rich lipoproteins results in an increase in HDL-C levels and a decrease in the levels of TG (134). These findings suggest that ASOs from CETP and ApoC-III may represent a promising therapeutic alternative.

#### Endothelial lipase inhibitors

Endothelial lipase (EL) knockout studies (135,136) and anti-EL antibodies (137) in mice demonstrated that inhibiting EL function resulted in increased plasma HDL-C levels. Furthermore, the overexpression of the human EL gene in mouse liver significantly reduced levels of circulating HDL-C and apoA-I (138). Loss-of-function mutations in EL expression have been observed to lead to increased levels of HDL-C and support the idea that inhibition of endothelial lipase may be an effective mechanism to increase HDL-C (139). In contrast, some studies point to an atherogenic role of EL, with a positive association between plasma levels of this enzyme and coronary artery calcification and inflammation (140,141).

### Liver X receptors (LXR) agonists

By coordinating the expression of target genes in various tissues, LXR agonists increase the flow of cholesterol from the periphery to the liver, where it is metabolized and excreted (142). Short-term administration of synthetic LXR agonist T0901317 in mice promoted reverse cholesterol transport in vivo from macrophages, at least in part, inducing an enrichment of HDL subclasses that enhance the plasma’s ability to promote cholesterol efflux by passive diffusion. and mechanisms mediated by SR-BI (143). In healthy subjects and hypercholesterolemic patients, reverse cholesterol transport pathways were similarly induced as in animal models by BMS-852927. However, an increase in plasma and liver TG, plasma LDL-C, apoB, apoE and CETP were also evident (144). In addition, there are potent synthetic LXR agonists, including GW3965, LXR-623, GW6340, AZ876 and ATI-111. LXRs play a central role in the regulation of ABCA1 expression in macrophages (145), and also induce expression of ABCG1 (146), thus increasing the formation of HDL mediated by these transporters.

In conclusion, despite evidence from observational studies, there are no indications to use HDL-C as a risk marker or even to consider increased plasma HDL-C as a therapeutic means to prevent CAD. In patients with very low HDL-C, diagnosis and intervention is important to exclude secondary causes and identify single-gene causes in anticipation of potential risks. Therapeutic approaches targeting HDL such as niacin, fibrates, and CETP inhibitors increased plasma levels of HDL-C but failed to reduce cardiovascular events. Therefore, we can say that it is insufficient to measure plasma levels of HDL-C as a way of estimating its atheroprotective function. Still, ways to increase cardiovascular protection with therapies aimed at HDL metabolism are not well established. Thus, further studies are needed to understand the mechanisms of molecular action and cellular interaction of HDL, enhancing the function of the HDL system.
